# Chronic pulmonary cavitary tuberculosis in rabbits: a failed host immune response

**DOI:** 10.1098/rsob.110016

**Published:** 2011-12

**Authors:** Selvakumar Subbian, Liana Tsenova, Guibin Yang, Paul O'Brien, Sven Parsons, Blas Peixoto, Leslie Taylor, Dorothy Fallows, Gilla Kaplan

**Affiliations:** 1 Laboratory of Mycobacterial Immunity and Pathogenesis, The Public Health Research Institute (PHRI) Center at the University of Medicine and Dentistry of New Jersey (UMDNJ), Newark, NJ 07103, USA; 2 Biological Sciences Department, NYC College of Technology, Brooklyn, NY 11201, USA; 3 Division of Molecular Biology and Human Genetics, University of Stellenbosch, Tygerberg 7505, South Africa

**Keywords:** cavitary tuberculosis, lung transcriptome, immune response, macrophages, *Mycobacterium tuberculosis*, rabbit

## Abstract

The molecular determinants of the immune response to *Mycobacterium tuberculosis* HN878 infection in a rabbit model of pulmonary cavitary tuberculosis were studied. Aerosol infection of rabbits resulted in a highly differentially expressed global transcriptome in the lungs at 2 weeks, which dropped at 4 weeks and then gradually increased. While *IFNγ* was progressively upregulated throughout the infection, several other genes in the *IFNγ* network were not. T-cell activation network genes were gradually upregulated and maximally induced at 12 weeks. Similarly, the *IL4* and B-cell activation networks were progressively upregulated, many reaching high levels between 12 and 16 weeks. Delayed peak expression of genes associated with macrophage activation and Th1 type immunity was noted. Although spleen CD4^+^ and CD8^+^ T cells showed maximal tuberculosis antigen-specific activation by 8 weeks, macrophage activation in lungs, lymph nodes and spleen did not peak until 12 weeks. In the lungs, infecting bacilli grew exponentially up to 4 weeks, followed by a steady-state high bacillary load to 12 weeks that moderately increased during cavitation at 16 weeks. Thus, the outcome of HN878 infection of rabbits was determined early during infection by a suboptimal activation of innate immunity and delayed T-cell activation.

## 2. Introduction

Tuberculosis (TB) caused by *Mycobacterium tuberculosis* (*Mtb*) is a major global health threat, with nearly nine million new TB cases and 1.1 million deaths annually [1]. When host protective immunity fails to control *Mtb* growth, as in about 10 per cent of infected immune-competent humans, progression to active disease occurs. In contrast, when immunity successfully controls the infection, as in about 90 per cent of infected individuals, no symptoms of disease are noted, and the bacilli are driven into latency [2], from which reactivation of active disease may occur later in life upon waning of host immunity [3,4]. The outcome of *Mtb* infection is determined by a complex immunological process, involving both the pathogen and the host [5]. Phagocytosis of inhaled *Mtb* by alveolar phagocytes activates a diversity of cell-signalling events, leading to the production and secretion of many cytokines, chemokines and receptor molecules that mediate the recruitment and activation of additional leucocytes. This cellular recruitment ultimately results in granuloma formation [6,7]. Well-differentiated granulomas in human pulmonary TB are characterized by central necrosis with caseation, followed by liquefaction, leading to cavitation that facilitates aerosol transmission of *Mtb* [8,9]. While the cells and soluble mediators of the host innate and adaptive immune responses responsible for controlling *Mtb* infection have been characterized [10,11], the exact molecular determinants underlying the failure of protective immunity are not fully understood.

We have established a rabbit model of pulmonary cavitary TB, which reflects many aspects of the human disease, as shown by similarities in lung histopathology and ability to develop caseation and lung cavities [12]. In this model, rabbits are infected with *Mtb* HN878 (a Beijing lineage strain) by the aerosol route, giving rise to chronic, progressive granulomatous pulmonary disease [12,13]. We and others have shown that chronic disease is driven by the continued survival of *Mtb* in infected macrophages, in association with ongoing recruitment of macrophages and lymphocytes into the enlarging granulomas [9,14,15]. Our previous reports have described the histological and microbiological characteristics of this infection model. However, the molecular determinants of the immune response that may contribute to failed protection from progression of infection to active disease have not been described.

In the present study, we used genome-wide transcriptional analysis to evaluate the immune response in the lungs of rabbits during the course of *Mtb* HN878 infection and the progressive development of pulmonary TB. We have adapted assays that rely on cross-reactive human reagents, and devised new methodology for monitoring rabbit immunity. In addition, the distribution of innate and adaptive immune cells and the organization of rabbit lung granulomas were studied. Our results suggest that the progression of *Mtb* infection to chronic granulomatous disease, culminating in cavitary pulmonary TB, is owing to impaired host-protective immunity, characterized by inefficient early activation of macrophages, delayed T-cell activation and a robust Th2 response.

## 3. Results

### 3.1. The transcriptomic response to *Mycobacterium tuberculosis* HN878 infection in rabbit lungs

**Figure 1.**
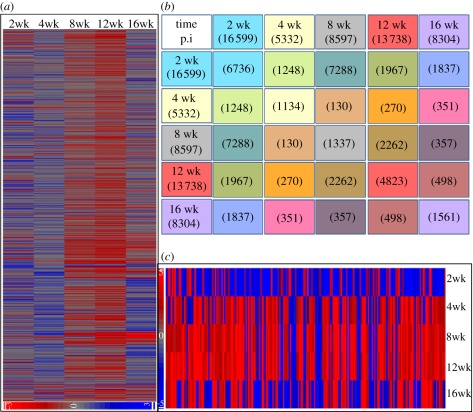
Global transcriptome of *Mtb*-infected rabbit lungs. (*a*) Intensity plot of rabbit genes expressed in the lungs at 2, 4, 8, 12 and 16 weeks of HN878 infection. (*b*) The numbers of unique and common genes differentially expressed at 2–16 weeks. (*c*) Intensity plot of the 209 commonly differentially expressed genes at all tested time points.

We have used whole-genome microarrays to examine gene-expression profiles in the lungs of HN878-infected rabbits at 2, 4, 8, 12 and 16 weeks post-infection. An intensity plot corresponding to the expression profile of all 43 603 probes in the Agilent rabbit array at each of the time points, relative to control (*T* = 0; 3 h post-infection) mRNA, is shown in figure 1*a*. Distinct changes in the global transcriptome over time were noted, with prominent shifts towards more upregulated genes from 4 to 8 and 8 to 12 weeks, and more downregulation from 12 to 16 weeks post-infection. Genes that were significantly differentially expressed at each time point were sorted based on a *p*-value of ≤0.05 and twofold change in expression, relative to control (figure 1*b*). Using this approach, genes that were significantly differentially expressed were enriched with a high level of confidence (*p* < 0.001; electronic supplementary material, figure S1). Already at 2 weeks, a large number of genes were significantly differentially expressed in response to HN878 infection; this number declined by 4 weeks post-infection. Thereafter, from 4 to 12 weeks post-infection, we observed a gradual but significant increase in both the total number of differentially expressed genes and the number of significantly upregulated genes in the lungs (figure 1*b*). This trend was altered at 16 weeks post-infection, at which time the gene-expression profile showed a general downregulation in comparison with previous time points, suggesting a shift in the host response to infection. A similar temporal pattern, with gradually increased numbers of upregulated genes from 2 to 12 weeks, followed by downregulation at 16 weeks, was seen in the common subset of 209 genes that were differentially expressed in all time points evaluated (figure 1*c*). The microarray data from these studies have been submitted to Gene Expression Omnibus (GEO; accession numbers GSE27992 and GSE33094).

To evaluate the functional relevance of rabbit genes expressed during HN878 infection, significantly differentially expressed genes were classified according to their biological roles. Annotation of the rabbit genome is currently incomplete, comprising roughly 2567 annotated genes. Moreover, as commercial microarray data analysis software does not include a rabbit gene database, we used human, rat and mouse homologues to assign functions to the annotated rabbit genes. Of the 2567 annotated input genes, the total number involved in all pathways examined ranged between 45 and 273. The top biological functional pathways modulated in response to HN878 infection are shown in table 1. The percentage of upregulated genes in the pathways associated with leucocyte migration, cell activation and adhesion, inflammatory response, cell death, leucocyte apoptosis, cell growth and proliferation, and cell movement gradually increased, with progression of infection from 2 to 8 and/or 12 weeks, and was reduced at 16 weeks post-infection. Of the genes significantly upregulated at 2 weeks, many were involved in the cell growth and proliferation, cell movement, and leucocyte migration and activation pathways. In contrast, the genes upregulated between 4 and 12 weeks post-infection were mainly involved in the inflammatory response and leucocyte apoptosis pathways. In comparison with earlier time points, a higher percentage of genes were downregulated at 16 weeks post-infection.

**Table 1.** Functional classification of differentially expressed genes in *Mtb* HN878-infected rabbit lungs (percentage), based on microarray analysis.

top biological function^a^	*p*-value	2 weeks		4 weeks		8 weeks		12 weeks		16 weeks	
		up	down	up	down	up	down	up	down	up	down
cell growth (149–179)	1.9×10^−44^ to 5.1×10^−41^	43	57	50	50	62	38	55	45	50	50
cell proliferation (179–224)	8.2×10^−59^ to 7.0×10^−48^	45	55	48	52	72	28	72	28	53	47
cell movement (181–189)	3.0×10^−61^ to 4.6×10^−55^	39	61	48	52	69	31	67	33	57	43
leucocyte migration (96–107)	1.2×10^−48^ to 1.7×10^−44^	38	62	53	47	71	29	67	33	57	43
cell activation (84–109)	3.0×10^−53^ to 4.2×10^−42^	38	62	48	52	69	31	72	28	51	49
cell adhesion (47–119)	1.4×10^−42^ to 6.2×10^−24^	36	64	48	52	75	25	72	38	54	46
cell death (61–273)	4.4×10^−55^ to 2.8×10^−27^	34	66	47	53	63	37	64	36	47	53
leucocyte apoptosis (45–55)	3.7×10^−38^ to 3.6×10^−24^	29	71	54	46	72	28	80	20	60	40
inflammatory response (129–168)	1.4×10^−54^ to 4.5×10^−38^	37	63	56	44	71	29	76	24	61	39

^a^Number in parenthesis represents total number of differentially expressed genes involved in a particular biological function.

### 3.2. Regulation of interferon-gamma, interleukin-four and T- and B-cell activation networks in HN878-infected rabbit lungs

**Figure 2.**
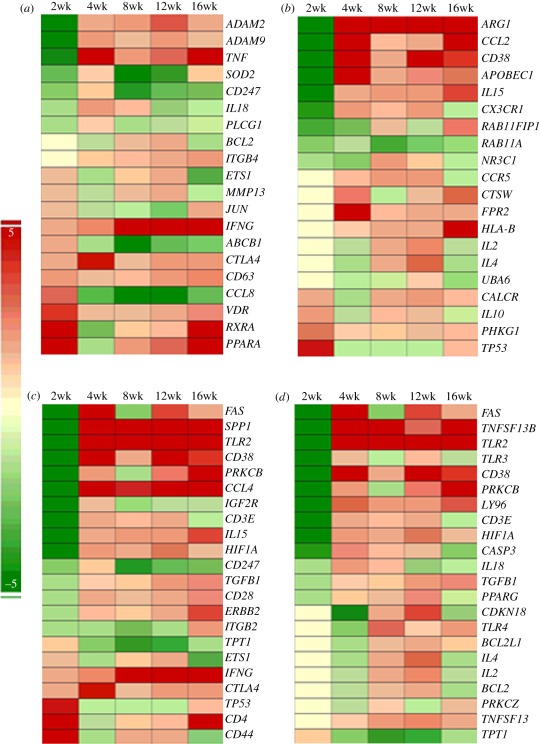
Expression kinetics of IFN-γ, IL-4, T- and B-cell activation network genes in HN878-infected rabbit lungs. Intensity plot of differentially expressed genes regulating or regulated by (*a*) IFN-γ, (*b*) IL-4, (*c*) T-cell activation and (*d*) B-cell activation networks from 2 to 16 weeks of infection. Red colour represents upregulation and green colour denotes downregulation. The intensity of respective colour is proportional to the level of expression.

To examine transcriptional changes in networks associated with host protective immunity during HN878 infection, we evaluated the differential expression of genes involved in the IFN-γ, IL-4 and T- and B-cell activation networks (figure 2). The IFN-γ network includes cytokines/chemokines—tumour necrosis factor alpha (TNF-α), IL-18, IFN-γ and chemokine (C-C) ligand 8 (CCL8)—enzymes and mediators of cell signalling. At 2 weeks post-infection, 11 of the 20 genes in this network were upregulated relative to controls. While *IFN-γ* expression continued to increase throughout the 16-week infection, the total number of upregulated genes decreased by 4 weeks, peaked at 8 weeks and then declined at later time points (figure 2*a*). With the exception of *IFN-γ*, which was highly expressed from 8 to 16 weeks, the majority of *IFN-γ*-associated genes were comparatively poorly induced. The 20 genes of the IL-4 network encode anti-inflammatory cytokines, such as IL-10, CCL2 and IL-15, and several transmembrane proteins and receptors (figure 2*b*). While only 4 of these genes were upregulated at 2 weeks, the number of upregulated genes increased to 11 (4 weeks), and 16 (8 and 12 weeks) decreased to 12 (16 weeks), as the infection progressed. Thus, the IL-4 network gene-expression profile suggested a gradual upregulation, with maximal expression in 6 of the 20 genes by 16 weeks post-infection. The T-cell activation network includes 22 genes encoding cytokines and chemokines, such as IL-15, IFN-γ, CCL4 and transforming growth factor beta (TGF-β), several receptors and cell-signalling molecules. Only 7 of the genes were upregulated at 2 weeks, while 15–17 genes were moderately upregulated from 4 to 16 weeks post-infection (figure 2*c*). High expression levels of lymphocyte activation marker (*CD38*), *TLR2*, macrophage inflammatory protein (*CCL4*) and osteopontin (*SPP1*) at 4 weeks that was sustained until 16 weeks of infection suggested an early and robust inflammation in the lungs in association with progression of the disease. Only one (*TPT1*) of the 22 genes in the B-cell activation network was upregulated at 2 weeks, as expected, as acquired immune response to *Mtb* infection is induced only by 3–4 weeks post-infection. However, 13 genes were upregulated at 4 weeks, and the total numbers and expression levels of genes in this network increased at 8 and 12 weeks (18 genes), with some of the genes showing the highest expression (5 genes) and some declining (12 genes) at 16 weeks of infection (figure 2*d*). Importantly, *TNSF13* (or APRIL) and *TNSF13B* (or BAFF), two key markers of B-cell homeostasis and proliferation [16], were upregulated in the lungs at 4 weeks post-infection. Expression of these genes was sustained to 16 weeks, suggesting a continued activation of B cells during infection (figure 2*d*). Similarly, the lymphocyte-activation marker CD38 was highly expressed from 4 to 16 weeks. Taken together, the expression pattern of the IFN-γ (Th1), IL-4 (Th2), T- and B-cell activation network genes suggests that a mixed Th1–Th2 response was induced in the lungs of HN878-infected rabbits. The interactions among the different genes, their properties and relative level of expression in each network, at 4 weeks post-infection, are shown in electronic supplementary material, figure S2.

### 3.3. Expression kinetics of selected genes associated with macrophage activation in HN878-infected rabbit lungs

**Figure 3.**
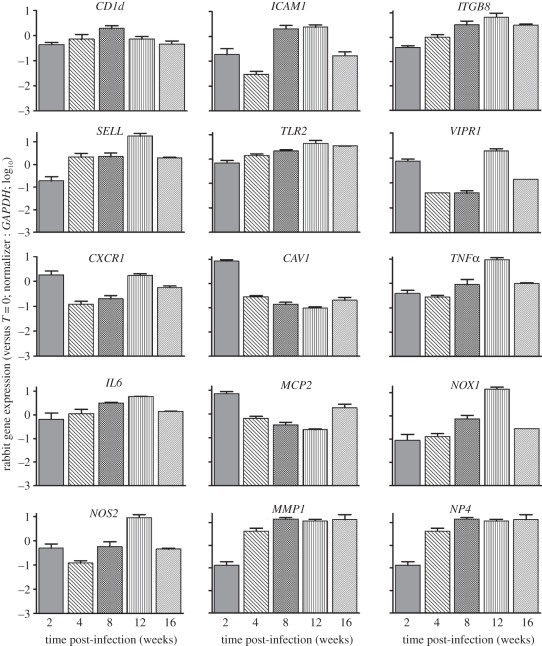
qPCR analysis of macrophage-activation genes in HN878-infected rabbit lungs. Genes encode for inflammatory cytokines (*MCP2*, *TNF-α*, *IL6*), cell surface receptors and membrane proteins (*ITGB8*, *CXCR1*, *TLR2*, *VIPR1*, *SELL*, *ICAM1*, *CAV1*, *CD1d*), antibacterial molecules and enzymes (*NP4*, *NOS2*, *NOX1* and *MMP1*). The values plotted were normalized to the transcript levels at *T* = 0. Each experiment was repeated at least twice in three to four biological replicates per time point.

To examine the expression patterns of genes associated with macrophage activation in the lungs over the course of infection, we used quantitative, real-time polymerase chain reaction (qPCR). The selected genes included the inflammatory cytokines *TNFα* and *IL6*; macrophage cationic protein-2 (*MCP2*) and C-X-C type receptor-1 (*CXCR1*); toll-like receptor-2 (*TLR2*), caveolin-1 (*CAV1*) and *CD1d*; host antibacterial molecules NADP(H) oxidase (*NOX1*), nitric oxide synthase (*NOS2*) and defensin (*NP4*); cell-adhesion molecules vasoactive intestinal peptide receptor-1 (*VIPR1*), intracellular adhesion molecule (*ICAM1*), selectin-L (*SELL*) and integrin β8 (*ITGB8*); and tissue remodelling and fibrosis (*MMP1*; figure 3). These genes showed three expression patterns: (i) progressive upregulation from 2 to 12 weeks post-infection with downregulation at 16 weeks; (ii) upregulation at 2 weeks with downregulation at 4 and 8 weeks post-infection; and (iii) gradual downregulation from 2 to 12 weeks, followed by moderate upregulation at 16 weeks post-infection (figure 3). *CD1d* expression was upregulated from 2–8 weeks and then downregulated at 12 and 16 weeks post-infection. Taken together, these expression profiles suggested reduced and/or delayed induction of molecules, associated with activation and maturation of the phagocytes, cell recruitment, inflammation and antimicrobial activity during the first 4 weeks of infection. Thereafter, macrophage activation, as indicated by gene-expression levels, peaked at 12 weeks and then declined.

### 3.4. Expression of Th1 and Th2 regulatory genes in the rabbit lungs during HN878 infection

**Figure 4.**
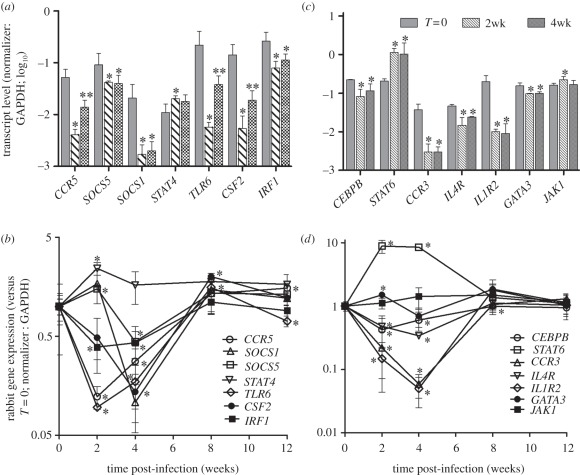
qPCR analysis of genes involved in the Th1- and Th2-regulatory response in HN878-infected rabbit lungs. (*a*,*b*) Genes that regulate the Th1-type immune response. (*c*,*d*) Genes that regulate the Th2-type immune response. The values plotted were normalized to *GAPDH* transcript for both (*a*) and (*c*). The values were relative to the levels at *T* = 0 for (*b*) and (*d*). Each experiment was repeated at least twice in three to four biological replicates per time point.

We next analysed the expression profiles of genes that specifically regulate the Th1 and Th2 type immune response by qPCR (figure 4). With the exception of *STAT4* (signal-transducer and activator of transcription protein-4), which was upregulated at 2 weeks, all Th1 genes examined were significantly downregulated as early as 2 or 4 weeks of infection, relative to controls, followed by moderate upregulation at 8 weeks post-infection (figure 4*a*,*b*). Among the Th2-regulatory genes examined, both *STAT6* (signal-transducer and activator of transcription protein-6) and *JAK1* (Janus kinase-1) were significantly upregulated at 2 weeks, and *STAT6* expression remained high at 4 weeks of infection. Other Th2 genes were downregulated at 2 weeks, relative to controls, and remained low at 4 weeks of infection (figure 4*c*,*d*). Expression of all Th2 genes evaluated stabilized at baseline levels at 8 and 12 weeks of infection. Overall, there appeared to be a profound early inhibition of several regulatory genes of the Th1 pathway in HN878-infected rabbit lungs, while inhibition of the Th2-regulatory genes was less profound.

### 3.5. Cellular composition of rabbit lungs during HN878 infection

To determine the immune cell composition at the site of infection, we enumerated single cell populations isolated from HN878-infected rabbit lungs using flow cytometry (table 2). The percentage of viable mononuclear cells decreased gradually from 71 per cent at 4 weeks to 24 per cent at 16 weeks post-infection. At 4 weeks post-infection, about 54 per cent of the total viable mononuclear cells were non-lymphoid and about 45 per cent were lymphocytes. This cellular distribution did not change significantly over the course of infection. About 60 to 82 per cent of the non-lymphoid cells were CD14^+^, identifying them as monocytes/macrophages (table 2). Of the total lymphocytes isolated from granulomatous portions of the lungs, the percentage of CD4^+^ T cells declined from 63 per cent (4 weeks) to 18 per cent (12 weeks), and then increased moderately to about 25 per cent of total lymphocytes at 16 weeks of infection. A slight reduction was also noted in the percentage of CD8^+^ T cells at 12 weeks, followed by a recovery at 16 weeks post-infection. A more than twofold increase in the percentage of CD4^−^/CD8^−^ cells in the total lymphoid population was noted from 4 to 12 weeks post-infection, and their proportion gradually increased from 4 (15%) to 16 (42%) weeks of infection. The majority of these CD4^−^/CD8^−^ cells appeared to be IgG^+^ B cells (table 2).

**Table 2.** Immune cell populations isolated from *Mtb* HN878-infected rabbit lungs (percentage), by flow cytometry (values are mean ± s.d.).

time post-infection	viable mononuclear cells	non-lymphocytes	CD14^+^	lymphocytes	CD4^+^	CD8^+^	CD4^−^/CD8^−^	B cells
4	71.0 ± 4.3	53.9 ± 3.4	78.1 ± 2.8	45.8 ± 3.4	63.1 ± 2.1	9.2 ± 0.9	27.6 ± 1.6	15.2 ± 0.9
8	49.3 ± 4.8	59.1 ± 4.1	60.2 ± 3.0	40.9 ± 4.1	33.5 ± 2.1	12.2 ± 1.2	50.9 ± 3.2	29.4 ± 2.2
12	28.4 ± 3.6	56.9 ± 1.3	64.0 ± 3.5	42.8 ± 1.3	17.9 ± 2.9	7.4 ± 1.5	71.4 ± 4.7	36.0 ± 4.3
16	24.4 ± 8.6	56.8 ± 5.0	81.9 ± 4.1	42.5 ± 5.0	25.6 ± 5.8	9.9 ± 0.7	59.8 ± 6.6	42.2 ± 2.5

### 3.6. Activation of tissue monocytes/macrophages in HN878-infected rabbits

**Figure 5.**
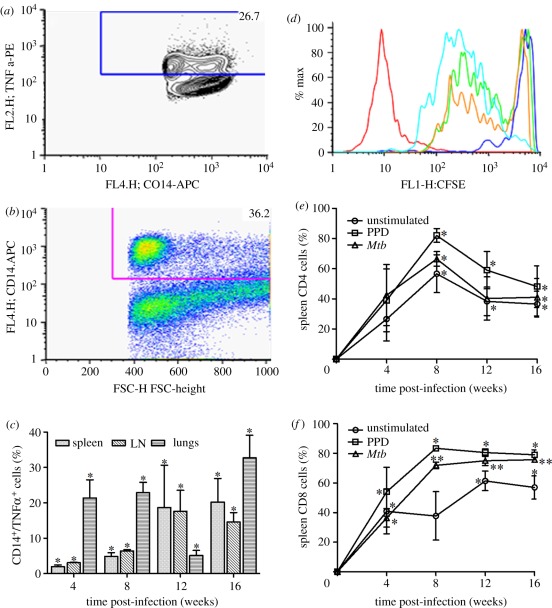
Activation of rabbit macrophages and T cells following HN878 infection. (*a*,*b*) Representative image of flow cytometry analysis of single cell suspensions from HN878-infected rabbit lungs after staining for markers of antigen-presenting cells (TNF-α and CD14). (*c*) Percentage of CD14^+^/TNF-α cells in the lungs, lymph nodes (LN) and spleen of HN878-infected rabbits. (*d*) Representative image of flow cytometry analysis of CD4^+^ T-cell proliferation in the spleen of HN878-infected rabbits. (*e*,*f*) Percentage of proliferating spleen (*e*) CD4^+^ and (*f*) CD8^+^ T cells either unstimulated or stimulated with PPD or *Mtb*.

We then assessed the extent of macrophage activation in the lungs, spleens and lymph nodes of HN878-infected rabbits by staining for CD14 and intracellular TNF-α and enumerating cell numbers by flow cytometry (figure 5*a*,*b*). We observed significant accumulation of CD14^+^/TNF-α^+^ cells in the rabbit lungs, compared with spleen and lymph node, at 4 and 8 weeks of *Mtb* infection (figure 5*c*). While the proportion of CD14 and TNF-α-expressing cells gradually increased in the spleen and lymph node from 4 to 12 weeks, a reduction in the same population was observed in the lungs at 12 weeks. However, the CD14^+^/TNF-α^+^ lung cell population at 16 weeks of infection was moderately higher than that of 4 and 8 weeks (figure 5*c*). Taken together, these findings are consistent with the kinetics of the gene-expression profiles seen in the lungs by the microarray analysis and suggest delayed optimal activation of monocyte/macrophages in the tissues of HN878-infected rabbits.

### 3.7. Analysis of cells of the adaptive immune response during HN878 infection of rabbit lungs

**Figure 6.**
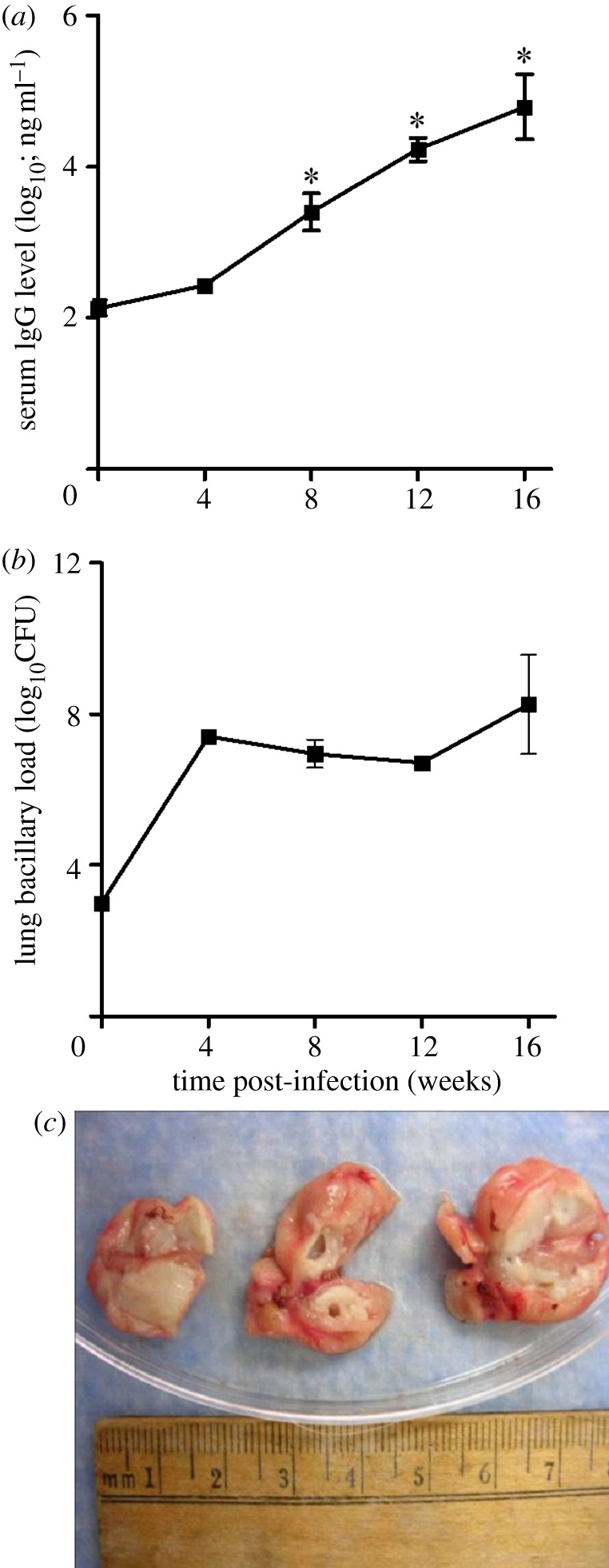
(*a*) Levels of anti-PPD immunoglobulin-G measured in the serum of HN878-infected rabbits. (*b*) The number of *Mtb* HN878 CFU in the infected rabbit lungs. (*c*) Cavitation of the granulomas with extensive necrosis noted at 16 weeks of HN878 infection.

Since cell-mediated immunity is crucial for the effective control of *Mtb* infection in humans and experimental animals, we examined the activation of CD4^+^ and CD8^+^ T cells. At early (2 weeks) and later (more than 12 weeks) time points, the relatively low numbers of T cells in the draining lymph nodes and lungs were insufficient for our functional assays. Thus, spleen cells from infected rabbits were stimulated *ex vivo* with purified protein derivative (PPD) or sonicated *Mtb* and their proliferation was evaluated by measuring carboxyfluorescein succinimidyl ester (CFSE) dilution using flow cytometry (figure 5*d*). The percentage of spleen CD4^+^ and CD8^+^ T cells that proliferated in response to PPD or *Mtb* stimulation was relatively low at 4 weeks and increased significantly at 8 weeks of infection (*p* = 0.01). While the proportion of CD4^+^ T cells proliferating *ex vivo* decreased at later time points, CD8^+^ T-cell proliferative capacity remained at the same level up to 16 weeks of infection (figure 5*e*,*f*). Unstimulated CD4^+^ and CD8^+^ T cells also proliferated, suggesting that endogenous antigen was driving some of the *in vitro* spleen T-cell response. Recent reports suggest a role for B cells in augmenting T-cell-mediated immunity against *Mtb* infection [17,18]. In addition, we observed upregulation of the B-cell activation pathway genes in rabbit lungs as early as 4 weeks of HN878 infection. To further explore the humoral immune response during HN878 infection of rabbits, we studied the extent of B cell activation by a novel serum, IgG ELISA, using PPD as the capture antigen. The serum concentrations of anti-PPD IgG were elevated at 4 weeks (2.2 log_10_ ng ml^−1^) and increased significantly up to 16 weeks post-infection (figure 6*a*), reaching over 5 log_10_ ng ml^−1^. These results were consistent with the increased IgG^+^ B cell counts in the infected lungs identified by flow cytometry (table 2).

### 3.8. Bacillary load and histopathology during progressive pulmonary tuberculosis in rabbit

**Figure 7.**
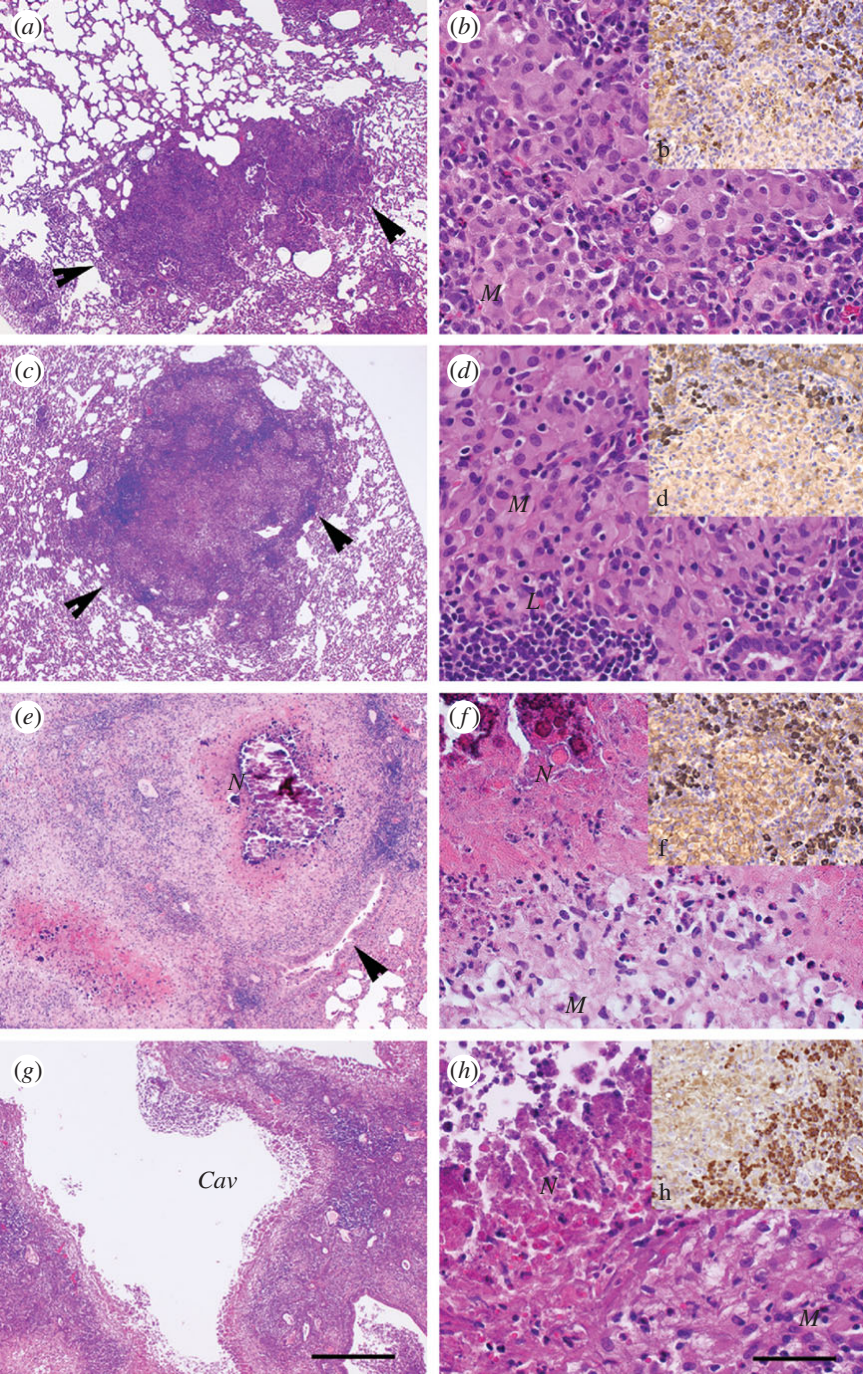
Histology of HN878-infected rabbit lung sections at 4 (*a*,*b* and b), 8 (*c*,*d* and d), 12 (*e*,*f* and f) and 16 (*g*,*h* and h) weeks post-infection stained by H&E (*a*–*h*) or by anti-IgG antibody for B cells (dark brown spots in b, d, f and h) and photographed at 4X (*a*,*c*,*e* and *g*) or 40X (*b*,b,*d*,d,*f*,f,*h* and h) magnification. Arrows in (*a*,*c*,*e*) show well-defined granulomas. *Cav*, cavitation; *M*, monocytes; *N*, necrosis; *L*, lymphocytes.

The lung bacterial burden, as evaluated by colony-forming unit (CFU) enumeration, demonstrated an early acute phase of HN878 growth (up to 4 weeks) followed by a chronic steady-state high bacillary load that continued up to 12 weeks post-infection. At 16 weeks, the time when cavities became apparent (see below), bacillary growth resumed (figure 6*b*). While the number of sub-pleural lesions did not change significantly from 4 (80 ± 20) to 12 weeks (88 ± 17) of infection, enlargement and differentiation of lesions were apparent (electronic supplementary material, figure S3). By 16 weeks post-infection, extensive necrosis and cavitation were seen in some granulomas (figure 6*c*). Haematoxylin and eosin (H&E)-stained sections of lung tissue at 4 weeks post-infection showed well-formed small granulomas composed of intermixed activated macrophages surrounded by lymphocytes (figure 7*a*,*b*). With time, the granulomas enlarged in size and, by 8 weeks, coalesced and became more organized, with foamy and epithelioid macrophages surrounded by a lymphocyte cuff and lymphoid aggregates (figure 7*c*,*d*). At 12 weeks post-infection, the lesions had enlarged further, macrophages appeared foamier and higher numbers of lymphocytes were present, mostly in the periphery of granulomas. Extensive central necrosis with extravasation of polymorphonuclear leucocytes and liquefaction were seen (figure 7*e*,*f*). Mineralization (calcification) was observed in some necrotic centres. By 16 weeks post-infection, some lesions had developed cavities, while others remained similar to 12-week lesions and a few showed signs of resorption (figure 7*g*,*h*). Since we observed progressively increased IgG production and accumulation of IgG^+^ B cells in the single-cell preparations, as well as upregulation of B-cell activation pathway in the lungs of infected rabbits, we examined the recruitment and distribution of B cells by immunohistology staining. Examination of lung sections at 4–16 weeks post-infection, by surface staining for IgG, revealed B-cell accumulation in the granulomas, predominantly in the lymphocyte cuffs, peripheral to macrophage-rich, necrotic centres (figure 7*b*,*d*,*f*,*h*). Interestingly, cavity formation in the lungs of the infected animals correlated with the downregulation of gene expression at 16 weeks of infection. Future experiments will address the link between the maturation of individual granulomas and the expression of marker genes of immune activation.

## 4. Discussion

Using a rabbit model of granulomatous cavitary pulmonary TB, we show that failure to control the progression of infection to cavitary disease was associated with delayed and suboptimal macrophage activation, and delayed differentiation and accumulation of antigen-specific T cells. Suboptimal activation of macrophages and T cells was associated with gradual upregulation of the *IFN-γ* and T-cell activation (Th1) gene networks. Thus, the early innate response was inadequate to control infection in the lungs, and extensive bacillary growth occurred before establishment of optimal T-cell activation. Once the infection was established, the combined innate, Th1 and Th2 type acquired responses maintained a high steady state of bacilli, with increasing lung pathology that progressed to cavitary disease by 16 weeks. Our histopathology analysis of infected lungs correlated with the findings from differential cell counts and gene-expression patterns during the acute (2–4 weeks) and chronic (8–12 weeks) phases of infection. For example, progressive accumulation of immune cells and their distribution during granuloma maturation, revealed by histology and single-cell analysis (4 weeks) of HN878-infected rabbit lungs, were consistent with a corresponding increase in the numbers and expression levels of genes involved in cell movement, growth and proliferation. Similarly, extensive necrosis and caseation of the lung granulomas at 12 weeks of infection were associated with reduction in viable mononuclear cell numbers and the expression of genes associated with cell death and inflammatory response. At 16 weeks (the time of cavity formation), the granulomas lost their ability to control bacillary growth (about 8 log_10_ CFU at 16 weeks), in association with a profound reduction in immune response gene expression.

A number of investigators have reported that the balance between Th1 and Th2 immune responses appears to be critical for the host defense against mycobacterial infection [6,19]. This balance is regulated by the levels of cytokines produced by the respective activated cells (i.e. IFN-γ by Th1 cells and IL-4 by Th2 cells) [20,21]. Pre-exposure of naive T cells to these cytokines is known to determine the direction of maturation of the effector cells: IL-4 and IL-13 drive T cells towards a Th2 phenotype, and IFN-γ drives the cells towards a Th1 phenotype [22–25]. Moreover, exposure of macrophages to Th1 and Th2 cytokines also affects their direction of activation [26]. Predominant exposure to IFN-γ activates the antibacterial activities of macrophages to effectively control *Mtb* [27,28]. In addition, macrophages activated by IFN-γ plus TNF-α produce inflammatory cytokines and chemokines, such as IL-6, IL-1β and chemokine (C-C) ligand 2 (CCL2), and bactericidal molecules, including inducible nitric oxide synthase (iNOS) and NADP(H) oxidase (NOX), which participate in the host defense against *Mtb* infection [29–33]. In contrast, elevated IL-4 production has been reported to block classical macrophage activation and favour the generation of alternatively activated phagocytes that are less efficient in controlling growth of intracellular bacilli [26,34]. These macrophages produce less iNOS and more arginase-1 (ARG1), peroxisome proliferator activated receptor gamma (PPAR-γ), chitinase 3-like3 (YM-1), macrophage receptor with collagenous structure (MARCO) and found in inflammatory zone (FIZZ), among other immune mediators [32,35,36]. Furthermore, while IFN-γ induces autophagy, which contributes to killing of intracellular *Mtb* in macrophages, exposure to IL-4 in the presence of IFN-γ abolishes autophagosome formation and its associated killing of *Mtb* via activation of a STAT6-dependent pathway [37,38]. Consistent with these reports, we observed early downregulation in the lungs of several genes involved in classical macrophage activation, and in the *IFNγ*- and *IL4*-activation networks. Furthermore, expression of several key host defense genes, including cytokines (*TNFα* and *IL6*) and bactericidal molecules, such as defensin-4 (*NP4*), *NOS2*, *NOX1* and *ICAM1*, peaked only at 8 and/or 12 weeks post-infection. It is of interest that the main Th2 transcription factor *STAT6* was upregulated during early infection, while a regulator of Th1 immunity, *STAT4*, was downregulated in the rabbit lungs at 4 weeks post-infection [39]. Our observations in the rabbit model of pulmonary cavitary TB are consistent with a mechanism in which Th1 activation is initially inhibited or delayed. This may explain the inability of the macrophages to control bacterial growth early during infection [40]. Control of *Mtb* infection has been shown to depend on the activation of antigen-specific T cells [8,21]. For example, antibody-mediated depletion of CD4^+^ T cells led to the reactivation of latent TB in the mouse [41]. Similarly, *Mtb*-infected CD4 gene knockout mice showed an increased susceptibility and altered immune pathology, emphasizing the requirement for CD4 T cells in protective immunity [42]. In addition, persistence of *Mtb* during the chronic phase of murine TB was shown to be owing to a suboptimal activation and reduced proliferation of antigen-specific CD4 T cells [43–45]. Similarly, CD8 T cells have been shown to participate in the protective immune response to TB, although their exact mode of action is less well understood [46]. For example, Turner *et al*. [47] reported an increase in the lung bacillary load in *Mtb*-infected CD8 knockout mice specifically during the chronic phase of disease. It has also been shown that while terminally activated memory CD8^+^ T cells recognize and lyse *Mtb-*infected macrophages, effector CD8^+^ T cells produce IFN-γ that would contribute to macrophage activation [48,49]. Moreover, reduced protective immunity against TB was observed in β macroglobulin knockout mice, which fail to produce functional CD8 cells, and in CD8-depleted mice and non-human primates, supporting an important role for these cells in host defense [50–52]. Furthermore, reactivation of TB in humans during anti-TNF treatment has been associated with reduced numbers and less efficient anti-mycobacterial activity of circulating CD8^+^ T cells in patients [53]. Taken together, these results support the prevailing view that CD4 and CD8 T cells are required for optimal protective immunity against *Mtb* infection. In the present study, we found that progressive T-cell activation peaked at 8 or 12 weeks of HN878 infection. However, this activation failed to protect the animals from progressive cavitary disease, perhaps owing to slow onset of the T-cell-mediated response.

In contrast to the clear contribution of T cells to protective immunity against *Mtb*, the role of B cells, mediators of humoral immunity, remains controversial [54–56]. Some studies have suggested that anti-TB antibodies produced during *Mtb* infection are not protective against active TB disease [57]. Consequently, it has been assumed that B cells do not contribute to the host protective immune response to this pathogen. However, recent reports describe contradictory findings on a potential role for B cells in the progression and containment of TB in mice [18]. Several studies demonstrated that the establishment of chronic TB and/or control of bacillary growth in *Mtb*-infected mice was unaffected by the absence of B cells, as shown in B-cell knockout animals [58,59]. In contrast, other experiments using B cell or Ig knockout mice showed more extensive bacillary growth in the lungs following *Mtb* infection [17,60]. Moreover, adaptive transfer of B cells to B-cell knockout mice, prior to *Mtb* infection, was reported to reconstitute the protective immune response to TB, resulting in lower bacillary numbers. In contrast to these observations, our results suggested that, in the presence of a T-cell response, concomitant progressive B-cell activation was associated with failure to control the acute infection and progressive granulomatous disease in *Mtb*-infected rabbits. Thus, our findings suggest that the outcome of HN878 infection in rabbits was determined by the kinetics of the host immune response and the sum total of immune activation of both T and B cells.

We and others have previously shown that various mouse strains infected with HN878 failed to mount a strong Th1 response compared with other *Mtb* strains such as CDC1551 and showed accelerated mortality in HN878-infected mice in comparison with infection by other *Mtb* strains [61–65]. Moreover, exposure of human monocytes to cell wall components of HN878 resulted in reduced production of pro-inflammatory cytokines in comparison to infection with CDC1551 [62]. Results of these studies suggested that virulence factors of HN878, including cell wall lipids such as phenolic glycolipid (PGL), are capable of subverting the protective immune response of the host via effects on macrophage activation, leading to high bacillary numbers and more severe disease [66]. In contrast with these studies, HN878 infection of C57BL/6 mice was reported to induce a strong Th1 response, which peaked early after infection and then declined sharply [67]. In the present study, we show that delayed macrophage activation at 2 weeks was associated with a lack of control of bacillary growth, and downregulation of immune activation at 16 weeks was associated with renewed growth of the bacilli in the lungs and cavity formation. This finding is similar to that in human TB cases, where cavitation of the lungs is associated with increased bacillary load. Our previous study suggested that, since T cells were absent from the luminal surface of the cavity, the phagocytes were more permissive for *Mtb* growth [9]. Taken together, our studies and those of others suggest that: (i) early macrophage activation is essential for the containment of bacillary growth; (ii) T-cell activation and IFN-γ production are required but not sufficient to control *Mtb* infection [68]; and (iii) B cells contribute to the lung immune pathology during active TB disease. The kinetics of these responses can shift the balance towards failed immunity versus protection. The results of these studies must ultimately be confirmed by comparison with the characteristics of a protective host immune response in rabbits infected with an *Mtb* strain that is successfully controlled. Such studies can facilitate improved understanding of protective immunity and the requirements for development of new intervention strategies to control TB.

## 5. Material and methods

Detailed procedures for the following can be found in the electronic supplementary material.

### 5.1. Rabbit infection

New Zealand White rabbits (Millbrook Farm, Concord, MA) were used for aerosol infection with *Mtb* HN878 as described earlier [13]. All procedures with *Mtb*-infected animals were performed in biosafety level 3 facilities, according to the protocols approved by the UMDNJ Institutional Animal Care and Use Committee. Bacterial loads in the infected lungs were evaluated as reported earlier [13]. Unless otherwise mentioned, all chemicals were purchased from Sigma (Sigma-Aldrich, Saint Louis, MO).

### 5.2. Total RNA isolation from rabbit lungs

Total RNA was isolated from rabbit lungs using TRIzol (Life Technologies, Carlsbad, CA) and processed with DNaseI and column-purified before subsequent use as mentioned earlier [13].

### 5.3. Rabbit whole-genome microarray analysis

Rabbit microarray experiments were performed with the arrays and reagents from Agilent Technologies as per the suppliers' instructions (Agilent Technologies, Santa Clara, CA). The microarray data processing and analysis were as mentioned earlier [69].

### 5.4. Quantitative real-time polymerase chain reaction

Total RNA from rabbit lungs was used in qPCR with gene-specific primers and SYBR green mix (Clontech, Mountain View, CA) as mentioned earlier [69]. The GAPDH transcript of rabbit was used for normalization of target genes. Fold change was calculated using the formula 2^−*ΔΔ*C^^*t*^. Each experiment was repeated at least 2 times with cDNA samples from 2–4 animals per group.

### 5.5. Preparation of single-cell suspension from spleen, lymph node and lung tissues

Rabbit lung sections were minced and incubated in collagenase and DNaseI. The spleen and lymph node segments were mechanically homogenized, and the homogenates were passed through a nylon cell strainer and centrifuged to collect the cell pellet. The erythrocytes in the cell suspension were lysed with acetate kinase (ACK) lysis solution and the isolated cells were counted after staining with trypan blue dye.

### 5.6. Flow cytometric analysis

Antibodies against human immune markers were screened for cross-reactivity against rabbit for these assays. Lung cells from rabbits were washed in FACS buffer (phosphate-buffered saline (PBS), 2% heat-inactivated foetal bovine serum and 10 mM NaN_3_), incubated in Fc block reagent (BD Biosciences, San Jose, CA), stained using FITC-anti rabbit CD4, PE-anti rabbit CD8 (BD Biosciences), Alexa 647-conjugated anti-human CD14 (cross-reactive to rabbit macrophages; AbD Serotec Inc, Raleigh, NC) or FITC-conjugated anti-rabbit IgG (AbD Serotec Inc) and biotinylated anti-rabbit IgM (BD Pharmingen, San Diego, CA, USA) followed by avidin-PE (BD Biosciences) for surface staining. For intracellular TNF-α staining, cells were incubated with Alexa 647 conjugated anti-human CD14 (cross-reactive with rabbit CD14), fixed with BD Cytofix solution and washed with Perm solution. Biotinylated anti-human TNF-α (cross-reactive with rabbit TNF-α; BD Biosciences) was added followed by avidin-PE. Flow cytometry results using FACSCalibur cytometer (BD Biosciences) were analysed using FlowJo software (Tree Star, Ashland, OR).

### 5.7. T-lymphocyte proliferation assay

Rabbit spleen cells were stained with CFSE dye, according to the manufacturer's instructions (Invitrogen, Carlsbad, CA). The CFSE-labelled spleen cells were stimulated with Concanavalin-A, heat-killed *Mtb*, PPD or left unstimulated. Cells were then stained for surface markers with anti-rabbit CD4 or anti-rabbit CD8 (BD Biosciences) followed by APC-anti-mouse IgG. Flow cytometry data were acquired as mentioned earlier.

### 5.8. Detection of serum immunoglobulin G by enzyme-linked immunosorbent assay

To measure the levels of anti-PPD-specific IgG in the serum of infected rabbits, we developed a new ELISA: 96-well plates (Corning, Lowell, MA) were coated overnight with PPD (Staten Serum Institute, Denmark; 10 µg ml^−1^ in PBS), washed with 0.025 per cent Tween 20 in PBS and blocked with 1 per cent bovine serum albumin in PBS at 37°C for 30 min. Serial dilutions of rabbit serum and polyclonal rabbit anti-PPD antibody (Antibodies-online GmbH, Atlanta, GA) were incubated in duplicate wells for 2 h at room temperature, washed and a 1:2000 dilution of alkaline phosphatase-conjugated goat anti-rabbit IgG (Southern Biotech, Birmingham, AL) in PBS was added for 2 h. Activity of alkaline phosphatase was detected using SIGMA*FAST p*-nitrophenyl phosphate solution according to the manufacturer's guidelines (Sigma-Aldrich).

### 5.9. Bacterial colony forming unit assay

Bacterial loads in the lungs of the infected rabbits were evaluated by plating 10-fold serial dilutions of the lung homogenates onto Middlebrook 7H11 agar plates (Difco, BD, Franklin Lakes, NJ). The plates were incubated at 37°C for 4–5 weeks. Colonies were counted, and results were expressed as number of CFUs in the whole lung.

### 5.10. Histology and immunohistochemistry of lung sections

Sections of formalin-fixed, paraffin-embedded lung tissues of infected rabbits were used for histology and immunohistology staining of B cells using an anti-rabbit IgG (Biocare Medical LLC, Concord, CA). Haematoxylin*-*Gill's formula was used to stain the nuclei (Vector Laboratories Inc, Burlingame, CA). Stained sections were photographed using a Nikon Microphot-FX microscope (Nikon Instruments Inc, Melville, NY).

### 5.11. Statistical analysis

The independent Student's *t-*test was used for statistical analysis (GraphPad software, La Jolla, CA). A value of *p* ≤ 0.05 was considered significant for all the experiments.
